# Career Choice and Career Change Among South African Health Professions: A Qualitative Study

**DOI:** 10.3390/healthcare14121775

**Published:** 2026-06-19

**Authors:** Modupe Busisiwe Makwarela, Christmal Dela Christmals, James Avoka Asamani

**Affiliations:** 1 Centre for Health Professions Education, Faculty of Health Sciences, North-West University, Potchefstroom Campus, Potchefstroom 2531, South Africa; christmal.christmals@nwu.ac.za (C.D.C.); asamanij@who.int (J.A.A.); 2 Health Workforce Unit, Health Systems and Services Cluster, World Health Organization-Regional Office for Africa, Cité du Djoué, Brazzaville P.O. Box 06, Congo

**Keywords:** career choice, career change, health professionals, health professions education, South Africa

## Abstract

**Background:** Despite being considered a country with a larger health workforce in Africa, the South African health workforce continues to experience shortages and a maldistribution of health workers across regions and sectors. Current projections suggest that the workforce is expected to decline further, especially among doctors, nurses and midwives, in large part, due to attrition—which could compromise the delivery of primary health and maternity services. These health workforce shortages and uneven distribution threaten the sustainability and effectiveness of health services in South Africa and drives the need to investigate the factors that may be influencing career choice and change decisions among health professionals in South Africa. **Methods:** A qualitative exploratory study, making use of purposive sampling and semi-structured interviews, was conducted to investigate the factors influencing career choice and change decisions among health professionals in South Africa. The participants were qualified health professionals in the fields of medicine, nutrition, pharmacy, nursing, and psychology working in the private, public, and academic sectors. Data was collected until saturation was achieved and then thematically analyzed using MAXQDA 24. **Results:** A total of 10 participants made up of three males and seven females were interviewed. These participants worked in different employment sectors with some having dual roles in private practice, public sector, and academia. The analysis revealed three major themes that capture the nature of and factors influencing career choice and career changes occurring in South Africa. The first theme related to factors influencing career choice (including altruism, family influence, personal experiences, financial/job security, academic achievement, career guidance, and opportunity for change). The second theme focused on career change dynamics (nature of career changes and career transitions occurring in the form of specialization, switching health professions, exiting health professions, adding non-health interests, and shifting focus areas). The third theme revealed factors influencing career change. These were categorized into personal and individual factors, workplace or job-specific factors, and administrative factors. This study has contributed to understanding the career choices and career changes taking place within the health professions in South Africa. It has also revealed a need for reforms in policy and practice for the current health professionals who have no intention of changing their careers while highlighting implications for future training of health professionals. Also, addressing the challenges of poor working conditions, lack of support, unemployment and placement delays, and other administrative barriers will help mitigate some of the issues leading to health workforce shortages and inequities in the South African context. **Conclusions:** The strongest motivator for choosing a career in health professions is the desire to care for others, while retention of the health workforce is challenged by personal, workplace, and administrative factors. Enhancing workplace conditions and support systems, implementing policy reforms, and minimizing administrative barriers is essential for achieving universal health coverage and sustaining a resilient health workforce in South Africa.

## 1. Introduction

According to the World Health Organization and World Bank [1], at least half of the people in the world do not receive the health services they need in terms of both quantity and quality. This is why world leaders and the global health community have committed to the attainment of universal health coverage (UHC) to ensure that all people have access to the health services they need, when and where they need them, without suffering undue financial hardship as a result of using the health services [2]. To attain this, there must be an equitable distribution of a competent health workforce equipped with optimal skills at the facility, outreach, and community levels, who are adequately supported and enjoy decent work [2].

Unfortunately, more than 70% of African countries face critical shortages and maldistribution of health workers, with 33 out of 47 countries identified globally as having high vulnerabilities and put on the WHO Support and Safeguards List in Africa, constituting one of the biggest challenges in achieving UHC by 2030 [3,4]. Even though sub-Saharan Africa’s health workforce increased significantly in the last decade, after adjusting for population growth, constrained ability to produce, employ, and retain an adequate number of health workers has been identified as the major challenge [5,6].

The health workforce in South Africa was estimated at about 0.45 million, making up 13% of the health workforce in Africa [3]. However, South Africa is one of four countries projected to experience a reduced stock of practicing health workers if business remains as usual, especially among nurses and midwives [7]. In addition to the increased need for more healthcare workers, South Africa still faces the huge responsibility of managing challenges like health worker shortages, which is expected to improve the performance of health workers and ensure an equitable distribution of health workers in all settings [8].

Though significant effort is being put into training more health professionals, the possibility of career change occurring is a reality that cannot be ignored or avoided [9]. Because the choice of a career is a significant decision that sustains individual living needs by generating income and satisfying other needs, such as passions and hobbies, it can significantly impact an individual’s life satisfaction and wellbeing [10]. This is why career changes can be expected when career choice motivations or needs are no longer being met.

Possible reasons for career changes are complex and multifaceted and may be linked to how the choice of pursing this career was made in the first place. A scoping review of the literature highlights several factors associated with career choice and change decisions, with frequent thoughts of leaving the health profession for different reasons, including temporal, physical, and mental wellbeing [11].

Although some information is available in the general literature regarding factors influencing career choice and change decisions, such as lack of future growth opportunities, working conditions, inadequate remuneration, etc. [12,13,14,15], there seems to be limited recent evidence among qualified health professionals in the South African context [16]. Understanding the nature, motivations, and mitigation factors related to career choice and career change from the lived experiences of current health professionals will be necessary to re-evaluate what changes may be necessitated in the training, education, employment, and retention of future health professionals. This paper therefore sought to clearly identify career choice and career change influences, explain the career change dynamics, and investigate any career change intention currently taking place in the South African health professions.

## 2. Materials and Methods

### 2.1. Study Design

This study followed a qualitative exploratory design. This design allows a researcher to gain comprehensive understanding of a phenomenon while also capturing pertinent characteristics closely linked to or explaining actual occurrences [17]. This design was considered the most appropriate for this study as it provides a comprehensive and in-depth understanding of career choice and career change, a phenomenon that is not extensively studied among health professionals especially in the South African context.

### 2.2. Participants and Sampling

Participants in this study were selected using purposive sampling and recruited using convenience sampling. The eligibility criteria for participating in this study were being a qualified health professional working for over a year as a medical doctor, nutritionist, pharmacist, nurse, or psychologist.

Over 25 health professionals from the chosen categories were invited to participate in the study, the aim of which was to gain insight from health professionals in as many employment sectors, e.g., government, private, academic, etc., however, due to busy schedules of most health professionals, as well as time constraints for data collection for a PhD study, only 10 health professionals were available. These included two medical doctors, one nutritionist, two pharmacists, two nurses and three psychologists.

An independent person (the researcher’s assistant) invited the health professionals to participate in the study by sending messages via various health professions platforms and contacting as many as possible whose contact details were available, as well as those with professional profiles (qualifications, contact details, etc.) easily accessible on the internet. The professionals who indicated willingness to participate were then contacted by the primary researcher via telephone and provided with additional information including the purposes and process of the study. They were also advised on the consent process and then a date and time for the interview was set at the convenience of the health professionals, with the option of a virtual or face-to-face meeting. Participants who preferred a virtual meeting were sent a Microsoft Teams meeting link with a password, while participants who opted for a face-to-face meeting chose a quiet and private location. This was either their consulting room or the primary researcher’s office. Participants were recruited and interviews were conducted until data saturation was achieved (when new interviews no longer yielded new insights into the topic of inquiry). Although saturation was achieved with the 9th participant, it was confirmed through one additional participant.

### 2.3. Data Collection

Semi-structured interviews were conducted using a semi-structured interview guide containing main questions and probing questions (see Table 1). Although this interview guide was not pretested, it had been pre-approved by the scientific and ethics committees of North-West University (NWU). The in-depth interviews (virtual or face-to-face) were conducted in English by the primary researcher and lasted about 45–60 min. Four of the interviews were conducted in the primary researcher’s office, three were conducted in the offices of the health professionals, and three were conducted virtually via Microsoft Teams with one of the participants located in another province. The responses were audio-recorded with permission from the participants and later transcribed. Data collection took place between March and June 2025 and was conducted by the principal researcher, a PhD student, and a qualified health professional in private practice and with experience in academia. Interviews were conducted until data saturation was determined when no new themes or sub-themes emerged from the latest interviews. A sample size of 10 was deemed appropriate because of the depth of information obtained from the experience of the health professionals as well as their vast engagement in diverse employment sectors and the depth of the semi-structured interviews. The data collected provided insight into the personal experiences of the participants but also knowledge gained through interactions with fellow colleagues. A sample size of 10 has also been shown in previous qualitative studies [18,19] to be sufficient in providing rich content, especially when rigour can be maintained [20,21]. Throughout the process of data collection, the researchers consistently reflected on and discussed perspectives and opinions of the participants with the understanding that their own thinking and interpretations may be influenced by their experiences, prejudices, and beliefs as researchers with health professional backgrounds. This reflexive strategy, along with restrictions to what the respondents said, was maintained to safeguard rigour and minimize bias in the conclusion.

### 2.4. Data Analysis

Braun and Clarke’s six stages of thematic analysis [22] were followed in analyzing the data collected. This process involved familiarization with data (stage 1) during which the primary researcher was fully immersed in a process of actively listening to the audio recordings of the interviews while reading and rereading the transcripts of each interview and making notes of the main elements highlighted in the discussions. The next stage was to formulate initial codes (stage 2). Here, using MAXQDA 24 (qualitative data management software), the primary researcher and another independent researcher with experience in thematic analysis individually coded three transcripts. To ensure rigour and minimize bias, a meeting was then set up for the primary researcher, independent co-coder, and one of the authors (a qualitative expert) to discuss and reach a consensus on the codes [23] to be applied. The next step (stage 3) was to identify initial themes emerging from the codes by grouping similar quotes into sub-themes. These sub-themes were reviewed repeatedly (stage 4) and then grouped, summarized, and categorized into more descriptive and defined overarching themes (stage 5). These themes and sub-themes were then compiled comprising suitable quotes extracted from the interviews, which are representative of the themes and sub-themes identified, and then reported (stage 6) under the overriding heading of career choice and career change among health professionals in South Africa.

### 2.5. Ethical Considerations

Scientific and ethical clearance were obtained from the Health Professions Education (HPEd) Scientific Committee and North-West University Health Research Ethics Committee (NEW-HREC) respectively (Approval Number: NWU 00042-24-A1). Goodwill letters were also obtained from the Health Professions Council of South Africa (HPCSA), Association of Dieticians in South Africa (ADSA), South African Pharmacy Council (SAPC), and South African Nursing Council (SANC).

Individual informed consent forms were administered and obtained from each participant, in accordance with the NWU processes. To preserve the privacy and confidentiality of participants, they were sent individual, password-protected meeting invitations and they were informed that participation in the study was voluntary and that they had an option to withdraw at any point in the study, without any consequences.

### 2.6. Trustworthiness

Trustworthiness of the research process and findings is measured by credibility, confirmability, dependability, and transferability and was ensured throughout this study [24]. Credibility was ensured firstly by interviewing qualified health professionals. Also, the first author (MBM) transcribed all the audio recordings verbatim and then three of the transcripts were checked against the recording by the independent researcher and by the second author (CDC). Confirmability was ensured through verifying that each participant’s perspective was reported accurately in transcription of interviews and in the outcome of the analysis. To ensure dependability, a detailed description of the methodology was recorded, reflexivity was maintained and data collection and analysis were concurrent. Analysis and coding were done by MBM and an independent co-coder, and they were reviewed by CDC and the third author, JAA. The use of audio recordings, individual meeting invitation links, an interview guide, and transcripts of recordings also contributed towards ensuring dependability. Due to the individual nature of responses to the questions probed in this study, as well as the uniqueness of participants interviewed, the findings may not be entirely transferable although the methodology, study design, participants (health professionals), and context of the study may be transferred under similar circumstances among health professionals in South Africa.

## 3. Results

### 3.1. Demographic Characteristics

The participants in this study included 10 qualified health professionals consisting of two medical doctors, a nutritionist, two pharmacists, two nurses, and three psychologists. There were three males and seven females who worked in a mix of private practice, public hospitals, and academic institutions as represented in Table 2.

### 3.2. Themes and Sub-Themes

The thematic analysis yielded three themes: factors influencing career choice, career change dynamics, and factors influencing career change (Table 3).

#### 3.2.1. Factors Influencing Career Choice

The participants indicated that their career choice decisions were influenced by a myriad of factors including a calling to care for people, family influence, personal experiences with health professionals, desire for financial/job security, high school grades and subject choice combination, opportunity to change career, and career guidance.

##### Calling to Care for People

The desire to take care of people was the strongest motivation to choose a career in health profession with about seven participants expressing in one way or the other that they felt “called” to care for others. Two of the participants—both psychologists—described this as a ‘calling’ that stemmed from their life experiences and spiritual beliefs.

*“It’s not just a career for me, it’s more of a calling. I felt God was calling me for something more than sitting in an office and pushing paper.”*

—*Psyc 2*

*“I think real life circumstances that one would experience or go through somehow would be confirming that you seem to have this as a calling.”*

—*Psyc 1*

Many of these participants expressed that their desire to make a difference in the lives of others and in their communities was also complemented by a sense of fulfilment they derived from serving others.

##### Family Influence

Seven of the 10 participants had been influenced by family members when choosing their careers. For example, both doctors indicated how a family member they admired or respected shaped their career choice decisions.

*“… the influence came from a cousin of mine, who I grew up with. He’s retired now. He was a medical doctor.”*

—*GP2*

*“My dad said “why don’t you become a doctor?… Yes, but I ended up there.”*

—*GP1*

For one of the participants, the influence was a loved one who was ill and needed healthcare services. Observing this need was the motivation she needed to become a Nurse.

*“My father was ill at that time, and you know, the future was not sure. So, he advised me that, why don’t you apply for nursing? And then I did apply for nursing.”*

—*Nurs 2*

##### Personal Experiences with Health Professionals

For some of the participants, their career choice decisions were informed by previous personal experience where they personally received healthcare services or had seen healthcare professionals in active service. The admiration and perception of respect and success they observed inspired their decision to pursue a career in the health professions.

*“I remember as a child, we used to go to the nearest healthcare centre that was there for minor ailments… and when I got there, the sisters, the way they would treat you, you would be treated well.”*

—*Pharm 1*

*“He was just this kind soft person, respected, modest, people with humility, and besides that, he was doing well, and he never showed off.”*

—*GP1*

It is worth noting that the way health professionals present themselves or the way they are portrayed can influence the desire to pursue a career in the health professions.

##### Financial Security

In some cases, the availability of a bursary or guarantee of receiving a stipend while studying and the hope of taking home a sufficient income was highlighted as the motivating factor to choose their career.

*“In my time, when you were doing pharmacy, bursary was guaranteed, as long as you were from the background, like disadvantaged, it was not negotiated.”*

—*Pharm 2*

*“we choose careers … that we think are fulfilling financially.”*

—*Psyc 3*

*“for me, my driving thing was taking home a lot of salary.”*

—*Pharm 2*

The desire for financial security is a significant influence for many health professionals, as shown by six out of 10 respondents indicating that they chose their careers because they believed it will provide them with some degree of comfort and the ability to provide for themselves and their dependents.

##### School Leaving Results and Subject Choice Combination

Four participants chose their careers because of how they had performed in the subjects they had taken at high school.

*“What made me is that I was like the best student in that time, it was called Home Economics, a subject with food and nutrition.”*

—*Diet 1*

*“And for me, I started while I was in high school to say, Okay, I want to be a health professional, because, you know the way we choose our subject in high school…yes, I was doing biology, doing physics, so that already was channelling us as kids to say, what you want to study.”*

—*Pharm 2*

Performance in school examinations (leaving exams), informed by specific subject choice combinations, was a major consideration for whether these practitioners could apply and be accepted into certain institutions to pursue their chosen careers. It was revealed that those who had taken up science-based subjects, such as biology, physical sciences, and mathematics, and passed were more likely to choose and be accepted into a career in the health professions.

##### Opportunity to Change Career

One of the participants chose their current career (clinical psychology) simply because she got an opportunity to change her career from the field she had previously studied.

*“So, after my first qualification, I had the opportunity to now venture into one of them. I had four, specialties, so I chose psychiatry. So that was how I ventured out of nursing completely into clinical psychology.”*

—*Psyc 3*

This indicates that sometimes people choose a health professions career just because the opportunity is available to them. This choice can be regarded as more opportunistic than intentional.

##### Career Guidance

One participant, a nurse, was advised to become a nurse by an educational psychologist who came to their school to provide career guidance and support.

*“We had an educational psychologist who came in to add to sort of support us through the career choices.”*

—*Nurs 2*

Of all the participants, this was the only participant to mention that career guidance provided by the school she attended was a part of her career decision-making process. This highlights the unfortunate reality that career guidance is not prioritized as an important component of the process of choosing a successful career path.

#### 3.2.2. Career Change Dynamics

##### Types of Career Change

The concept of career change was not foreign to the participants in this study. All 10 participants revealed that they had been exposed to career change either through their own personal experiences or the experiences of other health professionals who are close acquaintances. From their responses, career change can be grouped into three main types, namely, (1) specializing within the profession, (2) changing from one health profession to another health profession, and (3) changing from a health profession to something else outside health professions. The following section provides in-depth descriptions of the career change patterns as described by the participants.

##### Adding on/Specializing Within the Profession (Advancement)

In this type of career change, a health professional decides to acquire more specialized skills and knowledge that allows them to perform certain tasks that they may otherwise not have been able to perform with their original training. In illustrating this phenomenon, one of the participants, who is a medical doctor, said he decided to obtain a postgraduate qualification in occupational medicine to enable him concentrate on one area of medical expertise.

*“I did a postgraduate diploma in occupational medicine because I want to concentrate… so, I’ll still be doing general practice, but, you know, just zoning into the occupation medicine but it’s still part of medicine.”*

—*GP2*

##### From Health Profession to Health Profession

This type of career change is characterized by health professionals changing their mind from their original choice to pursue another profession within health professions. This type of career change is highlighted as very common especially for nurses who choose to pursue other careers like psychology. One of the participants, a psychologist, put it this way:

*“Yes, yes, I’ve got a couple of people that I know that have changed from their professions to psychology, they are mostly nurses for some reason, oh, yes I’ve got two nurses that I know and they became psychologists.”*

—*Psyc 1*

##### From Health Profession to Something Else

In other instances, the participants explained that some health professionals reach a point where they decide that they are no longer interested in pursuing a career in health professions. These quit the profession completely at different stages. For some it is a decision reached immediately after obtaining the health profession qualification as in the case of a medical doctor who quit soon after completing his degree:

*“No, he’s not practicing medicine. He never even practiced. He just qualified and left.”*

(*GP2*)

Some quit after a few years of work and many others make a decision after working for a long time. One of the participants (a medical doctor) told the story of a friend he had studied with who decided to quit medicine and pursue his passion for DJing:

*“There was a friend of mine, he’s in the US. I studied with him, and he kept on saying, no… I can’t do this…his passion was music. He’s running around the whole place you know, as a DJ and he’s a doctor.”*

—*GP2*

In another instance, a participant (female pharmacist) narrated how she had observed male colleagues quit pharmacy especially after their internship and community service.

*“I’ve seen a lot of my colleagues leaving pharmacy, yes, and especially the male pharmacists. Male pharmacists are mostly the ones that leave the profession immediately they finish their internship and comserve (community service).”*

—*Pharm 1*

##### Career Transition

Another form of career dynamics exists not as a complete change but rather a transition wherein a health professional decides to add other interests into their daily lives to the extent that this other interest becomes almost equal in terms of time allocation, or they choose to change their focus area from one specific function to another within the same profession. In some cases, these other interests become the primary vocation as they bring in additional income to the health professional and the health professional’s career becomes the secondary vocation. One health professional put it this way:

*“I am also trying to do other things on the side, just for multiple income streams, but I don’t see myself not being a medical doctor.”*

—*GP1*

An example of a shift in focus areas would be changing from clinical practice to research or academia. This type is quite fluid and not clearly defined as intimated by a participant, who is a male psychologist in private practice, academia, and public sector:

*“Yes… they have um, partially parked psychology, maybe parked clinical practice for research or for lecturing.”*

—*Psyc 1*

##### Career Change Intention

Of all the participants included in this study, four participants had no intention of changing their careers, three participants considered changing their profession, and three did not comment on their career change intentions. Those who had career change intentions mentioned reasons such as curiosity, age-induced lifestyle adjustments, and specialization. Regarding curiosity, the motive was the need for a challenge, although this need was not strong enough to lead to a career change. A female psychologist who had transitioned from nursing, now working in academia and private practice, narrated her experience:

*“I wanted to study engineering to challenge myself but why should I go back to school? I don’t want to go to long school, you know, lengthy periods of school. I wouldn’t want to go through that again.”*

—*Psyc 3*

Other reasons like age and specialization were highlighted in the context of professionals not seeing themselves doing the same career in their old age and feeling that it would be better to choose a career they believe is manageable at an older age. This was the case for a female pharmacist working in the public sector. She highlighted that doing the tasks required in her current career may not be sustainable into her later years.

*“I’ve had thoughts of leaving pharmacy, but not entirely. Suddenly I think I want to be a clinical psychologist. I don’t see myself dispensing at the age of 50.”*

—*Pharm 1*

The four participants who had no intention of changing their careers felt that they were doing something they always wanted to and felt it complemented their personality and other interests or passions.

*“It blends so well with my personality. Okay, also the other work that I do. I’m also a pastor and I’ve seen that it helps me in my work as a psychologist.”*

—*Psyc 1*

*“I never felt like that. I don’t see myself doing any other thing besides this. I am also trying to do other things on the side, just for multiple income streams, but I don’t see myself not being a medical doctor. my feeling is I’m going to work until maybe I drop dead.”*

—*GP1*

#### 3.2.3. Factors Influencing Career Change

According to data gathered from discussions with the participants, there are many factors that may influence career change decisions among health professionals. These have been summarized below and have been grouped into three main categories. They are personal- or individual-, workplace-, or job-specific factors and administrative factors.

##### Personal/Individual Factors

The inability to make sufficient money to meet one’s personal needs was identified as a motivating factor for changing careers, especially where the main reason for choosing the health profession career was financial security. This motivation to change careers was higher if the new opportunity provided better remuneration and was more aligned with their personal interests. Two participants explained it this way:

*“Others would leave nursing, or maybe their professions, because of greener pastures. And of course, getting more money.”*

—*Psyc 3*

*“they go and specialize so that they can make more money and also on something which at least they are more interested in.”*

—*GP1*

For others, a change in family dynamics, for example, marriage, having children, or elderly parents was the reason for a career change. This is because all these life adjustments have an impact on a health professional from a time and finance perspective. This was highlighted by one participant:

*“My children have grown and they’ve gone to university, so why should I kill myself by going to hospital? I can just work privately and just have money to pay the necessities, and I’ll be fine.”*

—*Psyc 2*

Personal disappointment regarding work expectations was also cited by participants as a contributory factor to career change among health workers in South Africa. Personal life demands or unrealized expectations leading to fatigue, burnout, or feeling overwhelmed were highlighted as some of the reasons for health professionals deciding that it is better to change their careers or shift their focus areas professionally.

*“I remember talking to one lady who is a psychologist, but who is not practicing, she said to me, when she was doing her internship, she got so overwhelmed, such that the following year she wanted to take a break. And it looks like the break became three years, five years, up to today, she’s not practicing.”*

—*Psyc 2*

*“The reason why people leave health professions, to go do business or other ventures, is because of the reality that this is actually not meant to make us rich, but to make us afford very basic and important things in life not to become rich.”*

—*Psyc 3*

In an attempt to seek fulfilment and validation, or even recognition, some health professionals decide to change their careers. It was highlighted that health professionals who never got to pursue their dream careers end up feeling frustrated due to restrictions or limitations in their current functions. This was explained by two pharmacist—one in the public sector and another in the private sector.

*“They wanted to study medicine and they didn’t get the opportunity, or maybe they feel that they are sort of caged, yeah, you’re restricted…I know so much, yet I can only do so much.”*

—*Pharm 1*

*“This is a boring profession. I’m not getting to see the patient. I’m not getting to understand what the patient is going through. I’m not part of the treatment plan.”*

—*Pharm 2*

The desire for freedom to choose what professional services to offer, work schedules, and also workplace processes may lead to career change decisions. The need for autonomy, independence, and ability to practice their profession without following rules set up by others was enough to make them change careers. According to a psychologist in private practice:

*“Many people love autonomy and they don’t want monotony and they want to grow and brand themselves. When you are operating within the government, it’s a vision that is set out already and you may choke literally when you find that you are following things this way and you have got your own style of doing things so when you branch out, for instance go to private, it’s because you want to really explore more about yourself, brand yourself, grow yourself as a brand and become who you really want to become.”*

—*Psyc 1*

*“The problem was, just when my requests were not being granted, then I felt like I can’t have a boss, I need to make my own spaza shop (small business) and basically be my own boss.”*

—*GP1*

*“For me, it’s about not being challenged, or you want to be on the other level, If I can be a prescriber, I can be the one who calls shots on this patient.”*

—*Pharm 2*

The desire for professional growth and autonomy is one of the key reasons for career change among health professionals in South Africa. The desire to carve niche areas for themselves and expand their knowledge and skills may lead to specialization or narrowing of one’s clinical or professional duties. Because this process may be difficult while working a full-time job, a health professional may then decide to quit their current occupation completely.

*“In clinical work, especially in public, it can be challenging to study because you see patients most of the time, but if you want to study and get finished, I think you’ll take longer.”*

—*Psyc 2*

The feeling of being stuck in a position with no growth opportunities in sight, after performing the same tasks for a while and not progressing professionally was the deal breaker for others. This was the case of the psychologist who left nursing after feeling like she had reached a career ceiling.

*“There’s not much development. I think I reached a ceiling in nursing. So there wasn’t much that I could do as far as I was concerned, so I needed more challenge.”*

—*Psyc 3*

Some participants indicated that they needed an environment where they could advance themselves if provided with opportunities for continuous learning and professional development. This was highlighted by a nurse:

*“The environment in which we are working, we don’t give people opportunities. People must be given equal opportunities to grow… I cannot go into the profession at 22 from an impoverished environment where I cannot even pay for my part time studies, and then for 10 years I’m not taken for any continued learning program.”*

—*Nurs 2*

##### Workplace Factors

Heavy workload was considered an important motivator of career change among health professionals in South Africa. The challenges highlighted by participants included long hours without rest, shortages, and juggling other administrative responsibilities, like stakeholder liaisons and retail or operational tasks, leave health professionals overworked and at risk of not performing their clinical duties such as patient care, diagnosing, prescribing, and educating efficiently.

This was the case especially among participants who worked in the public sector as intimated by at least five participants.

*“Nowadays, you go in at half past four, you leave tomorrow at eight o’clock in the morning. You’re still leaving patients; you have never slept. You’ve never rested, it’s busy.”*

—*GP1*

*“Especially working for the government. It’s very frustrating. Staff shortage, equipment, long hours, not being appreciated. We had an intern who died. He was sick and they said he must work.”*

—*GP2*

*“We don’t have supervisors for the sections, so I’m the one dispensing. I’m the one supposedly becoming advisor. I’m the procurement officer.”*

—*Pharm 1*

*“We treat, we sell. I must be honest; we sell certain things you don’t even have as a pharmacist that information.”*

—*Pharm 2*

Some health professionals, who have been over-exposed to certain aspects of their job such as long hours or a lot of bleeding, felt they needed a career change for their own emotional or mental wellbeing as well as for the safety of their patients.

*“Nursing hours were very long and very tiring, and when you come back home, you’re very tired…but I chose psychology because I was tired of blood, I was tired of the hospital, I was tired of patients, tired of sickness and all that.”*

—*Psyc 3*

*“And some of them, just burnt out. And that burnout it’s detrimental to our patients, because they miss some of the opportunities.”*

—*Nurs 2*

Secondly, poor relationships in the workplace result in some professionals changing their career in South Africa. Some felt discriminated against, disrespected, and undermined due to poor interactions among peers, inter-professionally, and with superiors.

*“I had a medical officer who was supposed to be my senior. Racist as hell, and he was sort of a gatekeeper probably, he wouldn’t teach you anything, So I was taught by the nurses; when you ask something, he’d say, what do you want to know? You’re supposed to know this. So, so I decided, no, let me leave this one alone.”*

—*GP2*

Older professionals sometimes had trouble with younger practitioners because of differing clinical techniques or opinions:

*“Most of the people that are quitting nursing are the older ones, like the ones who have been nurses for a long time. The main reason being that they cannot cope with this generation, this new generation.”*

—*Nurs 1*

While in other instances, some felt that others got special treatment and favours from superiors (sometimes based on their tribes or races).

*“They actually give me those days, which people don’t want, then you find I have something to do… No, you have to work. This is the decision, I’m your boss. So, during such times I’d resign.”*

—*GP1*

It was also highlighted that some workplace tensions arise because certain health professionals regard their knowledge or position to be of greater value than that of their colleagues and become difficult or unreasonable to work with.

*“The older ones are more receptive but the young ones say “you can’t tell me anything, I don’t remember meeting you in the corridors of Medunsa.”*

—*Pharm 1*

Thirdly, a lack of employee wellness programmes and other support systems to help health professionals cope with difficult working conditions results in career change. Four health professionals highlighted the lack of support structures or systems such as regular peer review, debriefing, case conferencing, and other employee wellness programmes, which help professionals either clinically or mentally, as factors that impact job satisfaction and influence career change decisions.

*“The mental wellbeing of future psychologists has to be taken care of because a lot of them pass into the profession without dealing with issues. So now, later on they would project on the clients and then they would become a danger to the clients themselves; so I think we need to intensify their wellbeing, maybe what we say emotional fatigue.”*

—*Psyc 1*

*“Remember, you are carrying these people, but there’s no job satisfaction. What do you do? You are supposed to help people, but now you yourself. Nobody cares for you.”*

—*Psyc 2*

Some health professionals felt that they were exposed to unnecessary risks (professional and legal), which may impede their ability to enjoy career longevity. For example, being expected to perform certain tasks for which they are not skilled or for which they do not have the necessary resources (medication, equipment, supervision) due to shortages and other operational challenges. Below is the detailed experience of two nursing participants:

*“I had a patient who I found that the baby had died in the clinic, and she was not supposed to deliver in the clinic. The husband said we are taking her to a specialist in Polokwane, she’s going to deliver in Polokwane. Then one day, when I was supposed to knock off, the husband came in with the wife. He said, “my wife is not going to Pietersburg (Polokwane). She’s going to deliver here in the clinic”. But I said, “No, I’m going to lose my license, because if she delivers in the clinic and something happens, then I’ll be responsible because this patient was at risk as she had a twin pregnancy”. he said, “no ways”. When I checked the woman, she was eight centimetres dilated, so I couldn’t send her home. And then the man said, “she’s going to deliver here, you’re going to deliver her”. And remember, I cannot leave that patient to go to the hospital when she’s eight centimetre dilated. I delivered those twins, and luckily, they both survived.”*

—*Nurs 2*

*“These days you can forfeit your pensions because of the things that are happening. I would rather just go, yeah, even if I wanted to retire at the age of 65 but I can’t…there’s no medicines. All the machines are not working. Everything is not there in the theatres.”*

—*Nurs 1*

##### Administrative Factors

Poor administration, especially in the public services sector, was another factor pushing health professionals to change their careers in South Africa. At least three health professionals highlighted that they were being managed by people who have insufficient knowledge of health processes and procedures. This was evident in policies or processes that do not support the delivery of certain clinical services. Many health professionals were also discouraged by the absence of monitoring and compliance, leading to systems disintegrating and standards dropping, as well as exposure to politics and bureaucracy at work. In some cases, a total absence of management and accountability, which eventually resulted in not knowing who to complain to, left the health professional desperate and frustrated.

*“They wanted to help the community, but politics, bureaucracy chased them away.”*

—*GP2*

*“The way things are being done, there’s no control. There’s no management.”*

—*Nurs 1*

*“So, the things we are procuring, even the processes, you know its so hard that we are trying to procure something for a patient now, who is in ICU, we want magnesium sulphate, then you go to finance then they won’t even understand, really, why do you need the magnesium sulphate immediately? Then you are told, ah, turnaround time for the requisition is 21 days But after 21 days, that’s when I would receive this, but I need it Now!”*

—*Pharm 1*

Secondly, prolonged unemployment and undesirable placements contribute to career change among health professionals in South Africa. Three health professionals felt that jobs were hard to find, either because there were no posts available or posts were available in locations they were not interested in. They also highlighted that those who had to wait a long time for placements (for internship or community service) found this discouraging after having trained for a long time. In the case of psychologists, there were interview processes that they had to undergo to gain admission into training to become qualified clinical psychologists. Sometimes, they were not selected and would not even know why. These frustrations then led them to consider changing their careers and pursue other more accessible professions.

*“When you look at the number of years in training, can you afford to go into a degree and then you go into an honours? Do you have the money to do that? and then you wait for an opening at Masters level, there are people who went for interview three times they did not get in.”*

—*Psyc 2*

*“Not now, but I used to, I wanted to study social work because of lack of opportunities for employment, back then. yes, so I have thought about that, but not enough to entertain or to pursue it, but it came to my mind and social work was like, okay, because it’s going to take me to where I want, to work with people.”*

—*Diet 1*

*“You can’t train for six, seven years, and then the next thing you come back, they say, No, we don’t have a job for you. Medicine used to be a noble profession, you know. So nowadays, even our minister tells us we are not special. Doctors are not special.”*

—*GP2*

## 4. Discussion

Choosing a career has been defined as a process typically involving locating promising vocational alternatives, collecting information about them, comparing them on the shortlist, and eventually choosing one to pursue [25]. Career choice is a subject of great interest as seen in recent international and cross-country studies as well as global trends. It is influenced by a buffet of factors that may be personally, socioeconomically, academically, lifestyle-, role model-, and prestige-driven [26,27]. As highlighted in this study, altruism (“calling to care for people”), family influence, personal experiences, financial/job security, academic achievement, career guidance, and opportunity for change are some of the key drivers of career choice. This is in agreement with studies done across the world, although most of these studies focused on students and not qualified health professionals [28,29,30].

Systematic reviews reveal that youth in collectivist societies are more influenced by family expectations while personal fulfilment and autonomy are of higher priority when it comes to identifying a career in more individualistic societies [31] and the findings in this study, when compared with global literature, point towards South Africa as being a culturally diverse country with both collectivist and individualistic tendencies.

Career change is explained as a subset of work role transitions, which may take place in the form of a change of employers or a change in one’s actual job or work role [32]. It has also been described as a movement across occupational boundaries [33]. This movement across occupational boundaries implies that when one changes careers, the new occupation requires different skills, daily routines, and even work environments from the present one [34]. Sometimes, however, transitions occur in the professional lifespan of an individual, which may be in the form of promotions or change in focus areas and may not necessarily be considered a career change.

When we look closer into the dynamics of career change occurring in South Africa, we also observe similar trends to previous global studies [9,35,36,37], where change occurs as a specialization within the profession, switching between health professions, or leaving the health professions space entirely. It is also evident that career transitions, which occur in the form of adding other interests or changing focus areas, are also possible factors that can eventually culminate in career change. In this study, some health professionals, who had taken on other non-health related interests, ended up exiting the health professions space because the push factors such as financial security and alignment with personal goals were stronger than the pull factor of helping people and making a difference.

Regardless of the type of change taking place globally, it is apparent that career change and the pace of career transition is increasing, with non-linear pathways becoming the norm [9] and change intentions being shaped by curiosity, lifestyle preferences, and dissatisfaction.

Similarly to previous studies, drivers of career change in the Soth African health workspace, which include burnout, challenging workplace relationships, career stagnation, and the lack of opportunities for professional growth, do not differ from the challenges observed globally [38,39,40,41,42,43]. However, for many health professionals who have no intention of changing their careers, there is a desperate need for reforms in policy and education to address the challenges of poor remuneration, poor working conditions, lack of support, unemployment/placement challenges, and other administrative barriers. This will help mitigate the issues relating to workforce shortages and inequities in the South African context. These recommendations are in agreement with local and international calls for reforms in training, career guidance, and occupation-specific support towards improving recruitment, retention, and satisfaction among health professionals [8,44,45].

This study has a few limitations worth noting. Firstly, only selected categories of health professionals were included, whose responses may not reflect those of other professionals in other categories. Secondly, due to the limited sample size reached at the saturation point, the responses obtained may therefore not be generalizable or conclusive to the South African health professions workforce. Thirdly, the selection of health professionals in one location and the recruitment process may introduce selection bias and the responses may have been influenced by familiarity with the primary researcher, them being a colleague and working in the same community. It is a recommendation that further in-depth studies, both qualitative and quantitative, be conducted among a larger and more representative sample of health professionals belonging to more professional categories. These future studies should seek to learn more about practical recommendations for policies and practices, as well as for training of future health professionals who are equipped not just clinically but also from an administrative point of view.

## 5. Conclusions

This study revealed that strong career choice influences, such as the desire to care for people, should be leveraged and encouraged and the weakest influences, such as career guidance, should be promoted not just as an entry influence but as a tool to guide and navigate career decisions throughout a health professional’s career lifespan. Although experiences and challenges may be similar for most health professionals, their commitment and love for their chosen profession often motivates them to find ways to channel career frustrations healthily, sometimes by choosing specialization or shifting their career focus. Exploring other personal (non-health) interests and passions as well as pursuing continued professional development should be promoted as positive outlets that may not change workplace or job-specific problems but may alleviate burnout and frustrations linked to exit triggers. To achieve universal health coverage targets, the recruitment and retention of satisfied and resilient health professionals is non-negotiable, and attention to the current systems, improving workplace conditions, and strengthening health workforce policy would be the best place to start.

## Figures and Tables

**Table 1:** Semi-structured interview guide.

No:	Main Question	Probing Question(s)
1	What influenced your choice of becoming a health professional?	Why did you choose or why are you choosing to study towards becoming a health professional? What attracted you into becoming a health professional?
2	What reasons have you heard given for a career change decision among health professionals?	What would make you change your career? If your current career is different from your first career, what made you change your career?
3	From your experience or observations from colleagues, what types of career change moves have you heard about?	What types of career changes are you aware of that are taking place among health professionals?
4	Share your perspectives on how professionals changing careers could influence health professions training in South Africa.	How should health professions training in South Africa be positioned to respond to the career change needs of the professionals in South Africa? What changes are necessary in health professions education to manage or mitigate the impact of career change?

**Table 2:** Demographic distribution of participants.

Profession	Gender	Age	Working Experience in Years	Private Practice	Public Sector	Academia
Medical doctor (GP1)	M	49	20+	√		
Medical doctor (GP2)	M	55	30+	√		
Nutritionist (NT 1)	F	52	25+			√
Pharmacist (Pharm 1)	F	48	25+		√	
Pharmacist (Pharm 2)	F	35	12+	√		
Nurse (Nurs 1)	F	60	35+		√	√
Nurse (Nurs 2)	F	62	35+		√	
Psychologist (Psyc 1)	M	47	15+	√	√	
Psychologist * (Psyc 2)	F	54	10+	√		
Psychologist * (Psyc 3)	F	59	15+	√		√

A * indicates that the participant has experienced career change.

**Table 3:** Themes and Sub-themes.

Theme	Sub-Themes
Factors influencing career choice	Calling to care for people Family influence Personal experience leading to admiration Financial or job security School leaving results and subject choice combination Opportunity to change career Career guidance
Career change dynamics	Types of change Career transitions Career change intention
Factors influencing career change	Personal/individual factors Workplace or job-specific factors Administrative factors

## Data Availability

The anonymized transcripts used and/or analyzed during the current study are not available due to confidentiality agreements with participants as descriptions of workplace settings may be useful in identifying the participant’s identity; however minimum dataset may be accessed from the corresponding author on reasonable request.
